# A Mixed Approach for Clock Synchronization in Distributed Data Acquisition Systems

**DOI:** 10.3390/s24186155

**Published:** 2024-09-23

**Authors:** Gabriele Manduchi, Andrea Rigoni, Luca Trevisan, Tommaso Patton

**Affiliations:** Consorzio RFX, Corso Stati Uniti, 4, 35127 Padova, Italy; andrea.rigoni@igi.cnr.it (A.R.); luca.trevisan@igi.cnr.it (L.T.); tommaso.patton@igi.cnr.it (T.P.)

**Keywords:** timing systems, FPGA, SoC, RedPitaya

## Abstract

Proper timing synchronization is important when data from sensors are acquired by different devices. This paper proposes a simple but effective solution for System on Chip (SoC) architectures that integrates a general-purpose Field Programmable Gate Array (FPGA) with a CPU. The proposed approach relies on a network synchronization protocol implemented in software, such as Network Time Protocol (NTP) or Precision Time Protocol (PTP), and uses the FPGA to generate a clock reference that is maintained in step with the synchronized system clock. The clock generated by the FPGA is obtained from the FPGA oscillator via appropriate fractional clock division. Clock drift is avoided via a software program that periodically compares the FPGA and the system counters, respectively, and adjusts the fractional clock divider in order to slightly adjust the FPGA clock frequency using a Proportional Integral controller. A specific implementation is presented on the RedPitaya platform, generating a 1 MHz clock in step with the NTP synchronized system clock. The presented system has been used in a distributed data acquisition system for fast transient recording in the neutral beam test facility for the ITER nuclear fusion experiment.

## 1. Introduction

Timing synchronization is important when data from sensors are acquired by different devices [1]. Such a situation occurs, for example, in laboratories where different Analog-to-Digital Converters (ADCs) acquire signals from different measurements, or in an Internet Of Things (IoT) system where geographically distributed sensors acquire physical quantities such as temperatures or air pollution parameters. If data derived from different measurement devices are not tagged with the correct time, it is not possible to derive a proper correlation in order to describe and understand the phenomena under analysis, such as the experimental measurements in the laboratory or the temperature evolution in a geographic region. Several techniques can be adopted to achieve timing synchronization depending on the nature and the location of the involved data acquisition devices. In a laboratory, clock distribution may suffice to drive the ADC sampling and ensure that data samples from different devices are consistent in time. For larger plants or a geographically distributed IoT, clock distribution is not feasible anymore and network synchronization protocols are used instead [2]. Network Time Protocol (NTP) [3,4] is a widely used protocol for synchronizing clock and time between clients and a clock server. The NTP clock server reads accurate Universal Time Coordinated (UTC) from an authoritative clock source such as an atomic clock or GPS. The precision of NTP can reach less than 1 ms in a Local Area Network (LAN) [5] and tens of milliseconds on a Wide Area Network (WAN) [6]. NTP is widely adopted in Programmable Logic Controllers (PLCs) [7] in order to tag data samples with absolute time and the precision in time of NTP is in most cases adequate with respect to the PLC data sampling rates, normally not larger than 100 Hz. When a more precise time synchronization is required, e.g., when dealing with high data sampling rates, Precision Time Protocol (PTP) can be used [8]. In the PTP protocol, slaves synchronize their clock to a master clock by exchanging timestamped messages and storing their send and receive times. Slaves compute their delay in time and update their internal times accordingly. The precision of PTP relies on two principal factors: (1) network quality and (2) hardware-assisted message timestamping. Indeed, the algorithm used by PTP to update slave times is based on the assumption that the communication time between the slave and the master is independent of the message direction. PTP-aware routers prioritize PTP network messages during routing in order to approach this condition [9]. Hardware-assisted message timestamping allows removing the time uncertainty that would otherwise arise if message timestamping occurred via software, due to the fact that the message must be processed by the Internet Protocol (IP) stack before being actually sent [10]. NTP is ubiquitous in computer systems and open-source solutions for PTP implementation, such as PTPd [11], are available for Linux systems, making network-based time synchronization feasible on most computer systems. White Rabbit, an extension of PTP relying on synchronous Ethernet, currently represents the most precise solution for clock synchronization [12].

A practical problem arises when the synchronization achieved in the system must be transferred to the connected hardware devices. For example, in order to perform synchronized data acquisition, an ADC device must receive, in addition to the input analog signals, a hardware digital clock that is maintained in step with the (synchronized) system time. We propose here a simple solution for System on Chip (SoC) architectures that provides the synchronization of a FPGA generated hardware clock with the system clock of the embedded processor, using a mixed (hardware and software) approach. A SoC is basically an integrated circuit that takes a single platform and integrates an entire computer system on it. The SoC components include a central processing unit, input and output ports, internal memory, and a configurable FPGA. SoC systems have made it possible to create a plethora of portable devices used in systems pertaining to the IoT and more in general on embedded systems. In our proposed solution, the embedded processor is used to achieve network-based synchronization and an FPGA module provides a clock whose frequency can be fine-tuned. The synchronization control loop is closed by a software process that periodically compares the system times with a timer register managed by the FPGA and adjusts the frequency of the clock generator accordingly, using a concept similar to the PTP clock servo algorithm [13]. The clock servo algorithm takes as input the timestamp messages and computes time offset adjustments. However, the slave clock cannot be simply and abruptly corrected by setting the free-running timer counter to a new value. If done this way, there would be time intermission or time-back scenarios, potentially hampering the proper behavior of time-based applications. The servo algorithm instead slightly adjusts the internal slave clock with the goal of achieving zero time difference between the slave and the master. The PTP specification does not define the servo part, but often Proportional/Integral (PI) time correction is performed. In a similar way, starting from the measured difference between the system time counter and the FPGA one, a PI regulator adjusts the frequency of the FPGA-generated clock in order to achieve zero time difference, reducing as far as possible the jitter in the generated clock.

The proposed solution has been implemented in a RedPitaya board [14] in order to synchronize ADC data sampling. This system has been used in a large physics experiment to achieve distributed and synchronized data acquisition. 

The rest of the paper is organized as follows:

- Section 2 describes the technique adopted to implement fractional clock division in the FPGA to allow fine-tuning of the generated clock;
- Section 3 describes how the FPGA module has been interfaced with the processor and the servo-like algorithm adopted to achieve synchronization;
- Section 4 presents the RedPitaya implementation for distributed and synchronized data acquisition;
- Section 5 describes the application of the RedPitaya implementation in a large physics experiment;
- Section 6 presents the final conclusions and future work.

## 2. Fractional Clock Divider Implementation

The FPGA component in the presented system is a clock generator that can be fine-tuned in frequency. We implemented it without using a Phase Locked Loop (PLL). Even if digitally controlled PLLs are available on different FPGA architectures, our pure digital solution implemented as a VHDL method has the advantage of being fully portable. In order to get a clock output at the requested frequency it is necessary to derive it from the internal FPGA oscillator at frequency *f*_*osc*_ and divide its frequency by a given quantity *d* that is normally not represented by an integer number. Fractional clock dividers produce an output clock whose frequency is, on average, equal to *f_osc_/d*′ where *d*′ is a sufficiently precise approximation of *d*. An important parameter in fractional clock dividers is their frequency resolution in Hz per count, also known as channel spacing. The higher the frequency resolution, the smaller the channel spacing, and the closer *d*′ to *d*. 

In the simplest fractional clock divider implementation, a *k*-*bit* counter is used and at every FPGA clock occurrence, the counter is incremented by an integer number that is equal to $\left\lfloor \frac{2^{k}}{d}\right\rfloor$, i.e., the integer part of $\frac{2^{k}}{d}$. An output clock strobe is generated every time the counter overflows and, on average, every $2^{k}$ clock cycles $\left\lfloor \frac{2^{k}}{d}\right\rfloor$ overflows occur. The average output clock frequency is therefore:

(1) $$f_{clck}=\left\lfloor \frac{2^{k}}{d}\right\rfloor 2^{-k}f_{osc}≅\frac{f_{osc}}{d}$$

In order to derive the channel spacing of this method we observe that, given d, the closest output frequency that can be achieved is obtained by summing $\lfloor \frac{2^{k}}{d}\rfloor +1$ instead of $\left\lfloor \frac{2^{k}}{d}\right\rfloor$. Therefore, the minimum frequency step is $f_{osc}2^{-k}$. Larger resolutions can be therefore achieved using a larger number of bits in the counter register. 

Another approach to fractional clock divider implementation is to consider the integer N such that N≤d<N+1 and use the FPGA oscillator to count N+1 for m times and N for M−m times, generating an output clock strobe at the end of every count [15] Given M and m, the total number of oscillator clock cycles for generating M output clock strobes are:

(2) $$N_{clck}=\left(N+1\right)m+N\left(M-m\right)$$

The average output clock frequency is therefore $\frac{f_{osc}}{\left(N+\frac{m}{M}\right)}$. In order to derive the frequency resolution in this case, it is convenient to rewrite the average output frequency as:

(3) $$f_{out}=\left(\frac{fosc}{N}\right)\left(\frac{1}{1+\frac{m}{NM}}\right)$$

Observing that in Equation (3) for *N* large enough, $\frac{m}{NM}$ is much smaller than 1, since for $x\ll 1,\frac{1}{1+x}≅1-x$, then:

(4) $$f_{out}≅\left(\frac{fosc}{N}\right)\left(1-\frac{m}{NM}\right)$$

From Equation (4) the channel spacing, in this case, can be approximated as

$$\frac{df_{out}}{dm}≅-\frac{f_{osc}}{N^{2}M}.$$

**Figure 1:**
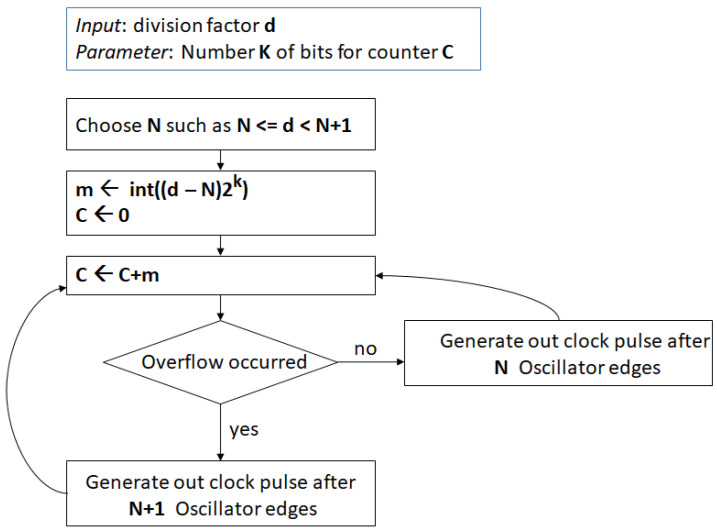
Fractional clock divider flowchart.

Assuming k bits for the cycle counter that must count up to *M*, the channel spacing turns out to be approximately $\frac{f_{osc}2^{-k}}{N^{2}}$, lower than in the former implementation. The drawback of this method is that the clock division for either N or N+1 can last for a large number of cycles introducing a periodic drift in the output clock frequency, even if maintained at the right average frequency. It is better to evenly distribute the clock division for either N or N+1, with the division by N+1 occurring with probability $\frac{m}{M}$. This can be easily achieved with a technique similar to the previous method, i.e., considering a k bits register and adding m at every cycle. When an overflow occurs, the clock division by N+1 is performed; otherwise, the oscillator clock is divided by *N*. The algorithm is summarized in the flowchart in Figure 1.

This algorithm has been implemented as a VHDL module that can be parametrized by the number k of bits for the cycle counter. The input ports for this module include the values N and m, and the divided clock is produced as output. The maximum jitter in time of the generated clock is $\frac{1}{f_{osc}}$. In the presented RedPitaya application using a Zynq 7020 SoC, $f_{osc}=125\;MHz\;$ and $f_{out}=1\;MHz$. The maximum jitter in time of the output clock is in this case 8 ns. The VHDL module also implements a 64-bit time counter that can be set via an external command to a given value and that is incremented at every output clock cycle. The current value of the counter can be read by the software and used to detect the drift with respect to the system clock to change the requested output frequency, i.e., the values of N and m, accordingly.

## 3. The Servo-like Software Application

The synchronization control loop is closed by a Linux process that periodically reads both the current system time and the current value of the time counter of the FPGA module. Based on the difference between the two counters, a new value of the reference frequency is computed and communicated to the FPGA module. The inputs of the FPGA module (parametrized by *k*, where $M=2^{k}$) are mapped against the following 32-bit registers:

- *N*: the value of the division;
- *m*: number of cycles with division by *N* + 1;
- *TimeInLo*: least significant 32 bits of the time value to be set;
- *TimeInHi*: most significant 32 bits of the time value to be set;
- *Command*: defines two bits. bit 0: take *m* and *N* inputs; bit 1: update Timer counter to the values specified by *TimeInHi* and *TimeInLo.*

In addition to the output clock, the actual value of the 64-bit time counter is mapped against two output 32-bit registers: *TimeOutHi* and *TimeOutLo*. These registers are read and written by the software process via Linux *ioctl*() calls. Both the time counters difference accumulated since the start of the process execution and since the last correction performed are computed and stored in variables *TotErr* and *StepErr*, respectively. At every iteration, occurring every 1 or 2 s, the requested clock period ${T^{\prime }}_{out}$ is computed starting from the previous ${T^{\prime }}_{out}$ value as follows:

$${T\prime }_{out}=T_{out}+K_{p}StepErr+K_{i}TotErr$$

**Figure 2:**
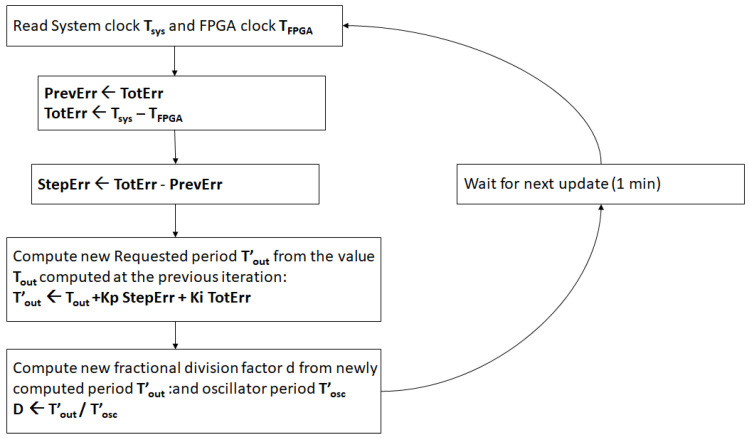
Servo-like flowchart.

The algorithm is summarized in the flowchart listed in Figure 2.

There is a similarity in the above computation with traditional PI control. *StepErr* can be considered an immediate measurement of the time error, while *TotErr* is the cumulative (integral) error since the start of the correction process. Indeed, we want to keep the frequency of the output clock in step with the system clock, but at the same time, we want to avoid long-term time drift. This is in particular true if we consider that periodically the system time can be updated by the network synchronization protocol (for NTP this occurs with a period ranging from 64 s to 1024 s). If only *StepErr* were considered in the correction, a difference between the system time and the FPGA time may persist. 

**Figure 3:**
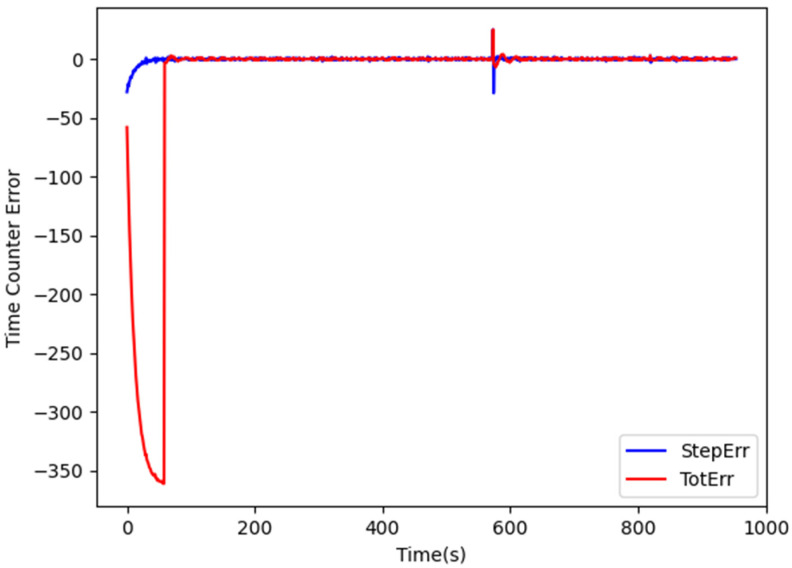
Evolution of TotErr and StepErr during clock synchronization.

In order to speed convergence, clock correction is performed in two phases. In the first one, only *StepErr* is considered, i.e., $K_{i}=0$, in order to tune the actual clock output frequency. When *StepErr* has decreased enough, the current system time is copied in the FPGA time counter and the correction starts taking *TotErr* in consideration. Figure 3 shows the evolution of *StepErr* and *TotErr* during the correction process. 

During the first 60 s, $K_{i}=0,$ and therefore *TotErr* is left uncontrolled, while *StepErr* is used to correct the FPGA clock frequency every second. Afterwards, the time counter is set to the current system time (i.e., *TotErr* is set to 0) and the update is performed every 2 s in order to give more time for accumulating errors. The values of $K_{p}$ and $K_{i}$ are set to 0.048 and 0.04, respectively. As shown in Figure 3, after a certain period during which *StepErr* and *TotErr* stabilize around 0, a sudden increase in their values occurs due to the adjustment of the system clock carried out by NTP. The performed correction then progressively adjusts the FPGA output clock so that *StepErr* and *TotErr* gradually approach 0 again, without any bump in the generated frequency and any discontinuity in the FPGA time count. When the new value ${T^{\prime }}_{out}$ has been derived, the parameters *m* and *N* are computed and transferred to the input registers via *ioctl*() calls. *N* is chosen such that $NT_{osc}\leq T_{out}<(N+1$)$\;T_{osc}$ where $T_{out}=1/f_{out}$ e and $T_{osc}=1/f_{osc}$ are the periods of the output and of the oscillator clocks, respectively. m is then set to the integer part of $M(\frac{T_{out}}{T_{osc}}-N).$

## 4. RedPitaya Implementation

A complete implementation of the presented synchronization mechanism has been carried out in the RedPitaya board. RedPitaya (RP) is an open-source platform that integrates fast and slow ADC and Digital-to-Analog Converter (DAC) and that can replace expensive and bulky instruments. The RP board has been considered for several reasons among which:

- Availability of high-speed ADC that can operate at a sampling speed up to 125 MHz;
- Zynq architecture. The Zynq SoC used in the RP board is well-suited for clock synchronization;
- Linux support. Linux is hosted in the ARM dual-core processor implemented in the Zynq 7020 SoC. Even if the lack of hardware-assisted network message timestamping prevents the use of PTP, the precision in time of the supported NTP synchronization is enough for our applications;
- Reduced cost, making the RP-based measurement much cheaper than other solutions equivalent in performance.

Clock synchronization has been integrated in an existing FPGA configuration for the RP board that allows both continuous and event-driven data acquisition [16]. In continuous data acquisition, acquired samples are streamed by the RP board via the network towards a data acquisition system. In this case, the sampling frequency is limited by the network and the data storage throughput, in practice limiting the maximum sampling speed to 1 MHz for sustained data streaming. In this case, the 1 MHz clock provided by the FPGA module can be directly used as the ADC clock, and the resulting data stream is possibly subsampled for lower data acquisition rates. In event-driven data acquisition, data are sampled during a time window centered around the event occurrence time. The event can be represented by an external trigger or by a condition met in the input signal, such as exceeding a given threshold. In order to acquire pre-trigger samples, a circular buffer is managed by the FPGA. In event-driven data acquisition, much higher sampling frequencies are normally used because this kind of acquisition is used to capture fast transient phenomena. Moreover, provided the rate of events is not too high, the acquired sample can be buffered in local memory, then sent over the network. In this case, the synchronized 1 MHz clock is still useful, even if an internal (non-synchronized) clock must be used to achieve higher sampling speed. The synchronized 1 MHz clock will be used now to timestamp the event occurrence times, ensuring that even in this case data from different devices are consistent in time, and the drift due to the internal clocks is normally negligible during the short acquisition time around the event occurrence time.

## 5. An Application Example

A direct application of this synchronization approach has been exploited within the nuclear fusion framework at the NBTF on a 1:1 Mockup of the MITICA experiment [17] (the full-scale prototype of the ITER [18] Heating Neutral Beam Injector). 

The Neutral Beam Injector is composed of a negative ion accelerator and a neutralizer so that the resulting beam of neutral particles can be used to heat the ionized gas (plasma) in a nuclear fusion experiment, without being distorted by the high electromagnetic field required to confine the high temperature plasma inside its container. In order to accelerate the negative ion beam, the ion source is kept at −1 MV potential in respect of the beam accelerator ground. Such a huge voltage requires a very accurate design in order to ensure electrical insulation. For this reason, a mockup of the ion source has been developed and it is currently under test. The main purpose of the MITICA Mockup is to address the voltage holding issues due to the one-of-a-kind stringent insulation requirements consisting in withstanding 1 MV in high vacuum on a single gap separating the Beam Source (up to −1 MV) from the Beam Source Vessel (BSV), at ground. 

**Figure 4:**
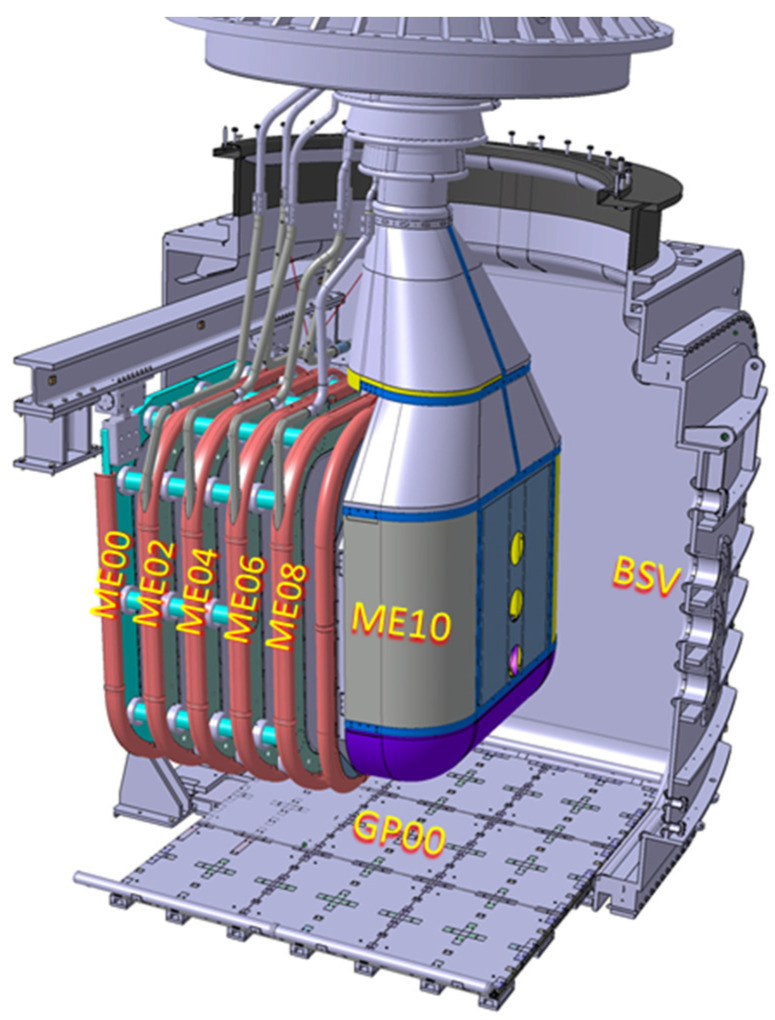
CAD view of the MITICA BS Mockup installed inside the MITICA Beam Source Vessel (BSV). MExx are the Mockup Electrodes at the different potentials from ground (ME00) to −1 MV (ME10) and connected to the High Voltage Bushing (HVB); the Ground Plate (GP00) is also visible on the bottom.

The Mockup (shown in Figure 4) reproduces almost faithfully the geometry of the external envelope of MITICA Beam Source: it is basically a set of six electrodes (MExx in Figure 4) biased from −1 MV (ME10) up to the ground potential (ME00) with 200 kV steps. In the Mockup system, an additional Ground Plate (GP00) placed on the bottom of the BSV is also present for diagnostics purposes. 

The Mockup system is also instrumented by a set of diagnostic systems: visible and infrared camera system, electrical measurement system, and a vacuum system composed of vacuum gauges and a residual gas analyzer. 

A reliable temporal correlation between the signals of such subsystems is a fundamental requirement to investigate and better understand the physics involved in such vacuum arcs and pre breakdown phenomena. In particular, the experiment is operated by pulses having duration up to hours, and within this time frame, signal drift must be carefully minimized. 

The electrical measurement system is based on RedPitaya board digitizers and the synchronization with each other and the other subsystems is based on the aforementioned mixed approach algorithm; the time base for the pressure signals and video cameras is based on NTP. 

The effectiveness of the mixed approach synchronization algorithm has been directly verified on the MITICA experiment with an ad hoc test as follows: a sine signal (Vpk = 5 V, f = 1 Hz) is delivered from a waveform generator to different RedPitaya boards for a time duration longer than 3600 s and the phase-shift is continuously measured, then at some specific times a pressure puff is generated inside the vacuum vessel and at the same time the amplitude of the sine signal is reduced to 0.5 V until the pressure puff is stopped. 

**Figure 5:**
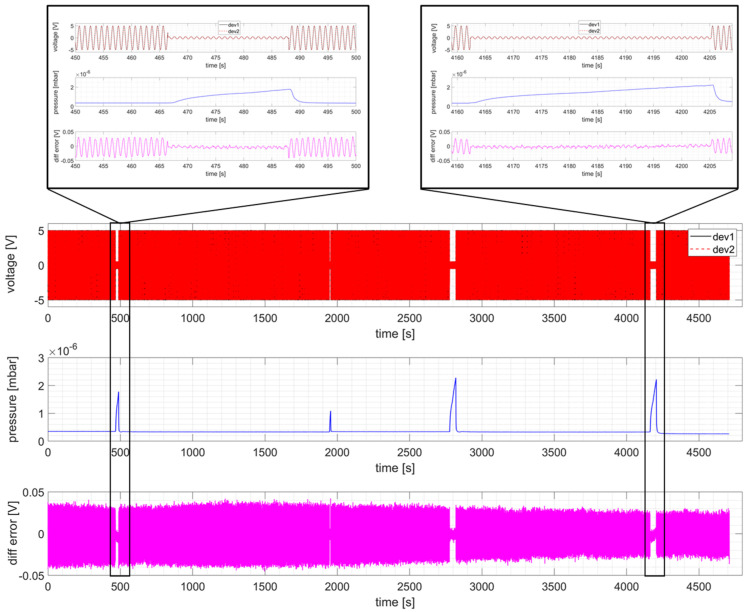
NBTF on-site test measurement during a dummy shot. Recorded voltage (Vpk = 5 V, f = 1 Hz sine) on two different RP devices (**top** panel), pressure measurement recorded on NTP device (**center** panel), and voltage difference between the two RP (**bottom** panel).

As shown in Figure 5, the phase shift between the sine signals recorded from the two RP devices is practically 0 (overlapped curves in top plot); a clearer picture is also provided from the difference of the two sine signals (third plot) whose absolute value is always below 0.05 V (εr < 1%) for the entire pulse, which means that no drift is observed. 

**Figure 6:**
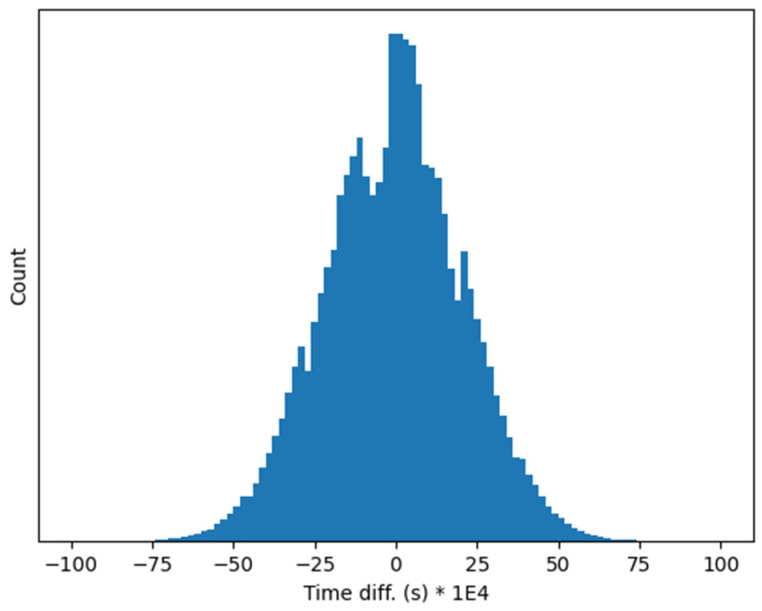
Relative time reconstruction error between two independent RP boards synchronized via NTP.

A more detailed measurement of the phase shift between the clock signals of the two different RP boards has been carried out by reconstructing the sampling time of every acquired sample. This is possible because the same and known sinusoidal signals is fed to both RP boards, and therefore it is possible to derive the sampling time difference from the sampled values in the two RP boards. The difference between the reconstructed sampling times is displayed in Figure 6, showing that the maximum time reconstruction error between the two RP board is in practice not larger than 7 ms. It is worth noting that this error is not related to the presented method, but to the NTP synchronization carried out in the two RP boards. We expect therefore that it will much reduced for a device implementing PTP synchronization.

In order to assess not only relative synchronization between the two RP boards, but also synchronization with other devices, the pressure puff acquired by a PLC using NTP time synchronization is displayed in the second plot of Figure 5, showing that the RP time base is well locked to the NTP time. 

## 6. Conclusions and Future Work

A simple solution for achieving hardware clock synchronization has been presented. In the proposed solution, the complexity of the FPGA part has been kept as simple as possible, implementing in practice a finely tunable fractional clock divider, closing the timing control loop by means of a servo-like software application. As a consequence, the proposed solution is much simpler in respect of other synchronization mechanisms such as the one proposed in [19] implementing directly in FPGA the PTP synchronization. In our case, the overall precision of the clock synchronization will depend on the chosen network-based synchronization mechanism and is independent of the presented application, whose purpose is to lock hardware clock generation to the (synchronized) system clock. 

SoC architectures represent the ideal configuration in this context because of the seamless integration between the processor and the FPGA. A complete implementation has been carried out on the RP board, making it possible to achieve synchronized data acquisition, both continuous and event-driven. The Zynq 7020 SoC used in the RP board does not allow hardware-assisted network message timestamping, and therefore the precise PTP synchronization cannot be achieved. Nevertheless, the precision in time of the NTP synchronization proved enough for the presented distributed data acquisition system in the ITER NBTF experiment. 

We are currently porting the clock synchronization mechanism to the KRIA board [20] for implementing a general-purpose timing generator for the generation of triggers and clocks to be used in distributed data acquisition systems. In this case, the UltraScale Zynq SoC of the board provides hardware-assisted network message timestamping, making the activation of the PTP network synchronization feasible and improving the precision of the timing board under development.

## Data Availability

Data are contained within the article.

## References

[1] Gore N., Lisova E., Åkerberg J., Björkman M. Clock Synchronization in Future Industrial Networks: Applications, Challenges, and Directions. Proceedings of the 2020 AEIT International Annual Conference (AEIT).

[2] Mahmood A., Exel R., Trsek H., Sauter T. (2017). Clock Synchronization over IEEE 802.11—A Survey of Methodologies and Protocols. IEEE Trans. Ind. Inform..

[3] Mills D.L. (1991). Internet Time Synchronization: The Network Time Protocol. IEEE Trans. Commun..

[4] Mills L. (1998). Adaptive hybrid clock discipline algorithm for the network time protocol. IEEE/ACM Trans. Netw..

[5] Giustina D.D., Ferrari P., Flammini A., Rinaldi S. Experimental Characterization of Time Synchronization over a Heterogeneous Network for Smart Grids. Proceedings of the 2013 IEEE International Workshop AMPS.

[6] Novick A.N., Lombardi M.A. Practical Limitations of NTP Time Transfer. Proceedings of the 2015 Joint Conference of the IEEE International Frequency Control Symposium & the European Frequency and Time Forum.

[7] (2008). IEEE Standard for SCADA and Automation Systems.

[8] (2021). IEC/IEEE International Standard—Precision Clock Synchronization Protocol for Networked Measurement and Control Systems.

[9] Burch J., Green K., Nakulski J., Vook D. Verifying the Performance of Transparent Clocks in PTP Systems. Proceedings of the Control and Communication 2009 International Symposium on Precision Clock Synchronization for Measurement.

[10] Wang W.-N., Li X.-C. Design and Performance Testing of a FPGA Based PTP System. Proceedings of the 2011 7th International Conference on Wireless Communications, Networking and Mobile Computing.

[11] Kovácsházy T., Ferencz B. Performance Evaluation of PTPd, a IEEE 1588 Implementation, on the X86 Linux Platform for Typical Application Scenarios. Proceedings of the 2012 IEEE International Instrumentation and Measurement Technology Conference Proceedings.

[12] Rizzi M., Lipinski M., Ferrari P., Rinaldi S., Flammini A. (2018). White Rabbit Clock Synchronization: Ultimate Limits on Close-In Phase Noise and Short-Term Stability Due to FPGA Implementation. IEEE Trans. Ultrason. Ferroelectr. Freq. Control..

[13] Maegawa R., Matsui D., Yamasaki Y., Ohsaki H. A Discrete Model of IEEE 1588–2008 Precision Time Protocol with Clock Servo Using PI Controller. Proceedings of the 2019 IEEE 43rd Annual Computer Software and Applications Conference (COMPSAC).

[14] Red Pitaya—Swiss Army Knife For Engineers. https://redpitaya.com/.

[15] Zhang S., Zhao C. Design for realizing arbitrary fractional divider based on FPGA wich duty cycle is up to 50%. Proceedings of the 2nd International Symposium on Computer, Communication, Control and Automation (ISCCCA-13).

[16] Manduchi G., Rigoni A., Trevisan L., Patton T. (2024). A versatile Board for Event-Driven Data Acquisition. Sensors.

[17] Toigo V., Dal Bello S., Bigi M., Boldrin M., Chitarin G., Grando L., Luchetta A., Marcuzzi D., Pasqualotto R., Pomaro N. (2019). Progress in the ITER neutral beam test facility. Nucl. Fusion.

[18] ITER Home Page. https://www.iter.org/.

[19] Pedretti D., Bellato M., Isocrate R., Bergnoli A., Brugnera R., Corti D., Corso F.D., Galet D., Garfagnini A., Giaz A. (2019). Nanoseconds Timing System Based on IEEE 1588 FPGA Implementation. IEEE Trans. Nucl. Sci..

[20] AMD Kria™ System-on-Modules. https://www.amd.com/en/products/system-on-modules/kria.html.

